# Identification of QTL for Grain Size and Shape on the D Genome of Natural and Synthetic Allohexaploid Wheats with Near-Identical AABB Genomes

**DOI:** 10.3389/fpls.2017.01705

**Published:** 2017-10-12

**Authors:** Lei Yan, Fei Liang, Huanwen Xu, Xiaoping Zhang, Huijie Zhai, Qixin Sun, Zhongfu Ni

**Affiliations:** ^1^State Key Laboratory for Agrobiotechnology, Key Laboratory of Crop Heterosis and Utilization, Beijing Key Laboratory of Crop Genetic Improvement, China Agricultural University, Beijing, China; ^2^National Plant Gene Research Centre, Beijing, China

**Keywords:** QTL, grain size, grain shape, synthetic wheat, D genome

## Abstract

Grain size and shape associated with yield and milling quality are important traits in wheat domestication and breeding. To reveal the genetic factors on the D genome that control grain size and shape variation, we conducted analysis of quantitative trait loci (QTL) using the F_2_ and F_2:3_ populations derived from a common allohexaploid wheat line TAA10 and a synthetic allohexaploid wheat XX329, which have near-identical AABB genomes and different DD genomes. Based on genotyping using wheat 660K single nucleotide polymorphism (SNP) array, TAA10 and XX329 exhibited 96.55, 98.10, and 66.26% genetic similarities of A, B, and D genomes, respectively. Phenotypic evaluation revealed that XX329 had higher thousand grain weight (TGW), grain length, width, area and perimeter than TAA10 across all environments, and the grain yield per plot of XX329 increased by 17.43–30.36% compared with that of TAA10 in two environments. A total of nine environmentally stable QTL associated with grain size and shape were mapped on chromosomes 2D and 7D and verified using near isogenic lines (NILs), with the synthetic allohexaploid wheat XX329 contributing favorable alleles. Notably, a novel QTL *QTgw.cau-2D* controlling grain weight was first identified from the synthetic allohexaploid wheat, which may be a more desirable target for genetic improvement in wheat breeding. Collectively, these results provide further insights into the genetic factors that shaped the grain morphology during wheat evolution and domestication.

## Introduction

Wheat was one of the first plant species to be domesticated and cultivated in the Middle East and was instrumental in spawning the agricultural revolution and the establishment of human civilization (Dubcovsky and Dvorak, 2007; Abbo et al., 2014). Domestication leads to the selection and spreading of specific phenotypic traits such as a non-brittle rachis to prevent spikelet shattering and soft glumes and hull-less seed for ease of threshing, which made wheat suitable for human planting and harvesting (Peng et al., 2011). Moreover, human intense selection also resulted in better agronomic performance and wide adaptability (Dubcovsky and Dvorak, 2007). For example, the development of high-yielding semidwarf varieties leads to the wheat “Green Revolution” in the 1940s (Hedden, 2003). The adaptability of common wheat to a large range of environments is partially due to the modification of vernalization and photoperiod requirements (Worland and Snape, 2001).

Grain size and shape, which are associated with yield and milling quality, are two of the most important traits in wheat domestication and breeding (Breseghello and Sorrells, 2007). Modern wheat varieties have higher grain width and lower grain length compared with ancestral wheat species which show greater variability in grain size and shape (Gegas et al., 2010). Larger grains could have a favorable effect on seedling vigor and promote yield increase (Gan and Stobbe, 1996). Geometrical models exhibited that changes in grain size and shape could result in increases in flour yield of up to 5% (Marshall et al., 1984). Therefore, genes or quantitative trait loci (QTL) associated with grain shape and size are of interest for domestication and breeding purposes (Simons et al., 2006; Williams et al., 2013).

Common wheat (*Triticum aestivum* L.) is an allohexaploid species with an AABBDD genome, derived from interspecific crossing between cultivated tetraploid wheat (*Triticum turgidum* L. AABB) and *Aegilops tauschii* (DD; Kihara, 1944; McFadden and Sears, 1946). Numerous studies revealed that in the D-genome donor to common wheat, *Ae. tauschii* provides a great source of genetic variability and improves agronomic traits (Dvorak et al., 1998; Mujeeb-Kazi et al., 2007; Reynolds et al., 2007; Rana et al., 2013). Accordingly, many synthetic allohexaploid wheat lines have been produced through hybridization of tetraploid wheat and *Ae. tauschii* and successfully used in wheat breeding (Xu et al., 2004; Francisc et al., 2007; Trethowan and Mujeeb-Kazi, 2008; Takumi et al., 2009). At the genomic level, several QTL analyses have been used to identify the D-genomic regions of synthetic allohexaploid wheat lines associated with grain size and shape (Okamoto et al., 2013; Yu et al., 2014). Notably, *Tg-D1* on chromosome 2D is *one* of well-known loci that have been recruited for the domestication of wheat grain size and shape. At allohexaploid wheat speciation, a dramatic change in grain shape occurred due to the mutation of the glume tenacity gene *Tg* (Kerber and Rowland, 1974; Nalam et al., 2007; Dvorak et al., 2012).

In 1964, the allohexaploid bread wheat TAA10 (cv Canthach, AABBDD) was hybridized to a tetraploid line of subsp. *durum* (cv Stewart, AABB), and the obtained pentaploid wheat (AABBD) was backcrossed by nine cycles to TAA10 as the recurrent parent, followed the pentaploid wheat was self-pollinated by three times to acquire the extracted allotetraploid wheat (ETW, AABB) containing the AABB component from TAA10. Afterwards, a resynthesized allohexaploid wheat XX329 (AABBDD) was produced by crossing ETW and the *Ae. tauschii* subsp. *strangulate* (line TQ18, DD), followed by genome doubling with colchicine (Kerber, 1964). The AABB subgenomes of XX329 should be very similar to those of the donor TAA10 due to the nine backcrosses. Thus, phenotypic variation in terms of grain size and shape was probably induced by differences on the D genome between the common wheat TAA10 and resynthesized allohexaploid wheat XX329 (Zhang et al., 2014). Here, we conducted QTL analysis for traits related to grain size and shape using the F_2_ and F_2:3_ populations derived from TAA10 and XX329 to identify D-genomic regions controlling grain size and shape variation. Furthermore, five near isogenic line (NIL) populations were developed to verify the environmentally stable QTL. These results provide further insights into the genetic factors that shaped the grain morphology during wheat evolution and domestication.

## Materials and methods

### Plant materials and field experiment

A common allohexaploid wheat line TAA10, the ETW, an *Ae. tauschii* accession TQ18 and the resynthesized allohexaploid wheat XX329 were grown with three replicates (two rows/replicate) in Shangzhuang, Beijing in the autumn of 2014. Seeds were well-distributed in rows that were 1.5 m long and 0.3 m apart with a sowing rate at 20 seeds per row, which is the same with the following field experiments.

An F_2_ population consisted of a number of F_2_ individuals as the progenies of the F_1_ individual by the cross between TAA10 and XX329, and an F_2:3_ population contained all the derived F_2:3_ lines from the corresponding individuals in the F_2_ population. For QTL analysis, a total of 328 F_2_ individuals generated by the cross between TAA10 and XX329 were grown in the greenhouse of China Agricultural University in 2014, and the derived 328 F_2:3_ lines were grown in Shangzhuang, Beijing in the spring of 2015. In addition, the other six F_2_ populations by the same cross and two parental lines were grown in the experimental field under six different environments at three locations (Shangzhuang, Beijing, E116°, N40°; Shijiazhuang, Hebei, E114°, N38°; and Linfen, Shanxi, E111°, N36°) in the spring of 2 years (2015 and 2016). These six F_2_ populations contained 377 (Beijing2015), 206 (Beijing2016), 265 (Heibei2015), 216 (Heibei2016), 198 (Shanxi2015), and 260 (Shanxi 2016) F_2_ individuals, respectively. The parental lines TAA10 and XX329 were grown with three replicates (two rows/replicate) in each environment.

Field trials for evaluating the plot yield of TAA10 and XX329 were performed in a randomized complete block design with three replicates (20 rows/replicate) at two locations (Beijing and Hebei) in the autumn of 2015.

### Phenotypic evaluation

The measurements of thousand grain weight (TGW), grain length (GL), grain width (GW), grain area (GA), and grain perimeter (GP) were performed using the grain analysis program developed by Wanshen Science and Technology Ltd. (Hangzhou, China; Cheng et al., 2015). For TAA10, ETW, TQ18, and XX329, seeds were harvested with 30 plants (10 random plants in each replicate). For each F_2_ and BC_4_F_2_ population, every individual was harvested and measured for these grain traits, and for the F_2:3_ progeny, all traits were described by the mean values of 20 plants for the corresponding line from each F_2_ individual.

In plot-yield trials of two parental lines TAA10 and XX329, spike length (SL), spikelet number per spike (SLN), spike number per plant (SN), grain number per spike (GN), and TGW were evaluated with 30 plants per replicate, and the data from SL, SLN, and GN were collected from the main tillers. SL was measured from the base of the rachis to the tip of the terminal spikelet, and SLN contained the fertile spikelet number and sterile spikelet number per spike. All grains in a single plot were collected for measurements of yield per plot (YPP).

The statistical analyses containing Student's *t*-test, variance analysis and correlation analysis were performed with SPSS version 20.0 (SPSS, Chicago, USA).

### SNP genotyping

Wheat 660K SNP array was designed by Chinese Academy of Agricultural Sciences and synthesized by Affymetrix (http://wheat.pw.usda.gov/ggpages/topics/Wheat660_SNP_array_developed_by_CAAS.pdf). Two parental lines TAA10 and XX329 were genotyped with wheat 660K SNP chip by Compass Biotechnology Company (Beijing, China). The genetic position information of SNP markers was provided by Compass Biotechnology Company (Unpublished data).

### DNA extraction

Genomic DNA was extracted from leaf tissues in the seeding stage using the cetyltrimethyl ammonium bromide (CTAB) method (Allen et al., 2006). The enough leaf sample of each plant was collected and stored at −20° to ensure obtaining DNA with high quality for genotyping analysis. Extracted DNA was dissolved and preserved in TE buffer, and the quality of DNA was assessed using 1% agarose gel electrophoresis.

### SSR marker development and analysis

The genomic sequence of *Ae. tauschii* is a useful reference for marker development on the D genome of wheat. The scaffold sequence published at ATGSP (http://aegilops.wheat.ucdavis.edu/ATGSP/)was used to identify the SSR region containing at least 10 dinucleotide or trinucleotide repeats, and primers for this region were designed in the flanking sequence using Primer3 web (version 4.0.0; http://bioinfo.ut.ee/primer3/). The synteny analysis of these SSR markers, *Ae. tauschii* markers and rice genes referenced to Luo et al. (2013), and the information of rice genes were obtained from the International Rice Genome Sequencing Project (Matsumoto et al., 2005). Published primer sequences for the SSR markers are available at http://wheat.pw.usda.gov/. The PCR was conducted in a 10 μL reaction system containing 50 ng genomic DNA, 1 μL 10 × reaction buffer, 0.2 μL 10 mmolL^−1^ dNTPs, 2.0 μL primer, 1 U rTaq DNA polymerase (Takara, Dalian) and 4.7 μL ddH_2_O. The PCR program was set to denature the template DNA at 94°C for 5 min, followed by 35 cycles at 94°C for 30 s, 55°C for 30 s, and 72°C for 30 s, and finally extend the PCR products for 10 min at 72°C. The length polymorphism of the SSR markers was identified using 8% non-denatured polyacrylamide gel electrophoresis (PAGE; Marklund et al., 1995).

### Linkage map construction and QTL analysis

For quick scanning of the entire genome to find best possible QTL, single marker analysis by a simple linear regression model were performed with Windows QTL Cartographer version 2.5 (Wang et al., 2012). The trait values and genotypic data of each marker in different populations were used for single marker analysis, and a significance level of 0.05 was used to declare that the marker was associated with the corresponding trait. Genetic linkage maps were constructed with the program JoinMap 4.0 (Van Ooijen, 2006). Markers with <5% missing data were employed and organized into linkage groups with the LOD thresholds ranged from 4 to 10. The order of markers in each linkage group was arranged using a regression mapping algorithm (Stam, 1993). The map distances were calculated based on recombination frequencies using the Kosambi mapping function (Kosambi, 1943). The QTL analysis using the composite interval mapping (CIM) method were performed with Windows QTL Cartographer version 2.5 (Wang et al., 2012). Model 6 with forward and backward regression was employed to QTL mapping. Five markers as cofactors and a 10-cM scanning window were chosen for the detection of QTL. The LOD threshold was set via 1000 permutations at *P* ≤ 0.05 (Yan et al., 2006). Considering the fact that phenotypic data of each genotype in F_2_ populations may be inaccurate for the quantitative trait, significance for QTL was defined as a LOD value higher than 2.0. The identified QTL were named according to McIntosh et al. (2011).

### NIL population development

To develop the NILs of the three QTL regions 2DS, 2DL, and 7DS, the F_1_ individuals derived from TAA10 and XX329 were backcrossed with the recurrent parent (TAA10). Marker-assisted foreground selection was performed in each backcross generation. The BC_4_F_1_ individuals which exhibited heterozygosity in each QTL region were self-pollinated to produce their corresponding BC_4_F_2_ populations. In addition, a total of 60 SSR markers on the D genome were used for background selection of the BC_4_F_1_ individuals. Finally, the numbers of BC_4_F_2_ populations for QTL verification on 2DS, 2DL, and 7DS were 1 (BC_4_F_2_-2DS), 2 (BC_4_F_2_-2DL-1 and BC_4_F_2_-2DL-2) and 2 (BC_4_F_2_-7DS-1 and BC_4_F_2_-7DS-2), respectively. The population sizes of BC_4_F_2_-2DS, BC_4_F_2_-2DL-1, BC_4_F_2_-2DL-2, BC_4_F_2_-7DS-1, and BC_4_F_2_-7DS-2 were 213, 231, 186, 150, and 153, respectively. These BC_4_F_2_ populations were grown in Shangzhuang, Beijing in the spring of 2017.

## Results

### Phenotypic evaluation

**Figure 1:**
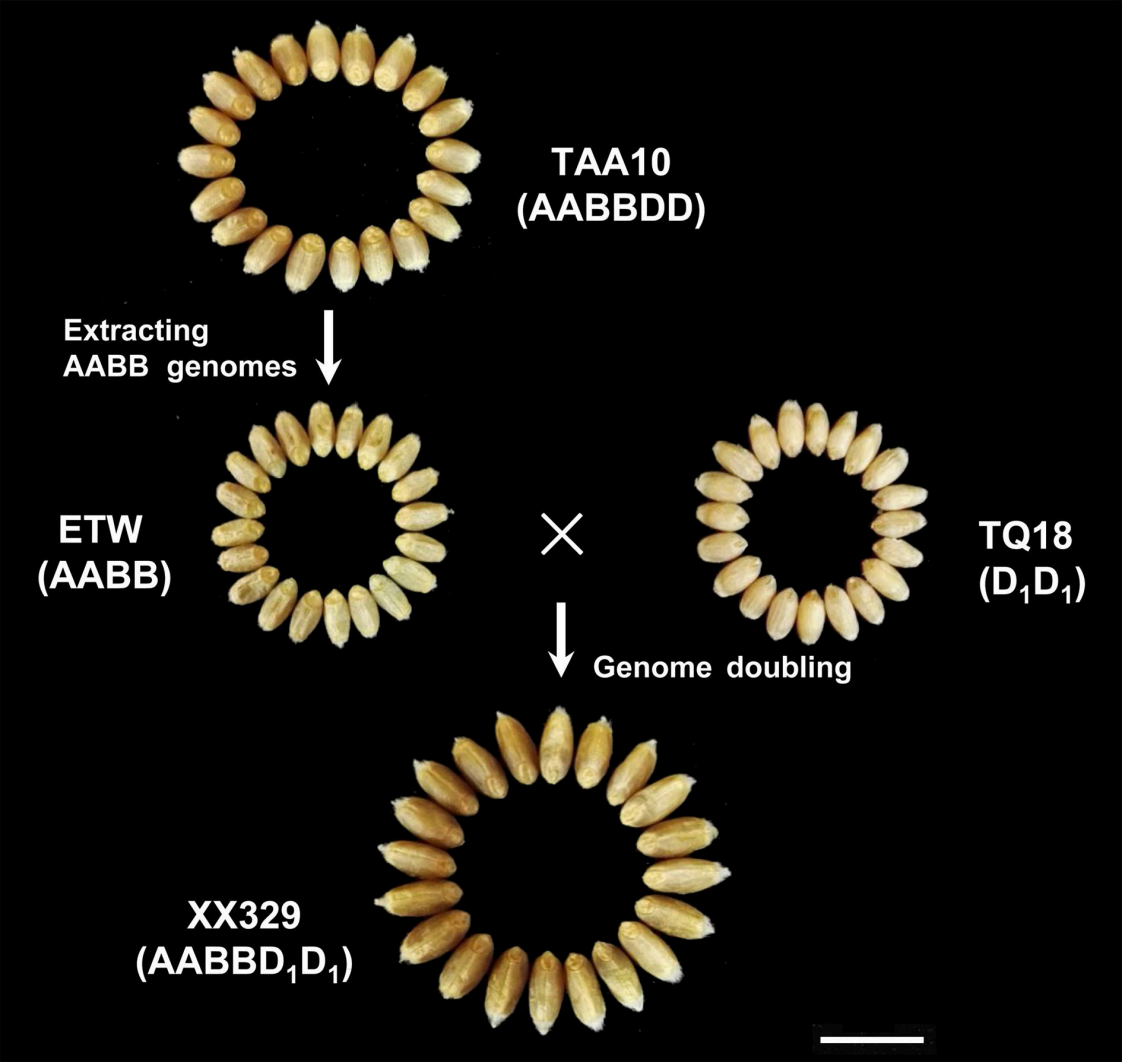
Grain morphology of the common allohexaploid wheat TAA10, the extracted allotetraploid wheat ETW, the *Ae. Tauschii* line TQ18 and the resynthesized allohexaploid wheat XX329. The scale bar represents 1 cm.

**Figure 2:**
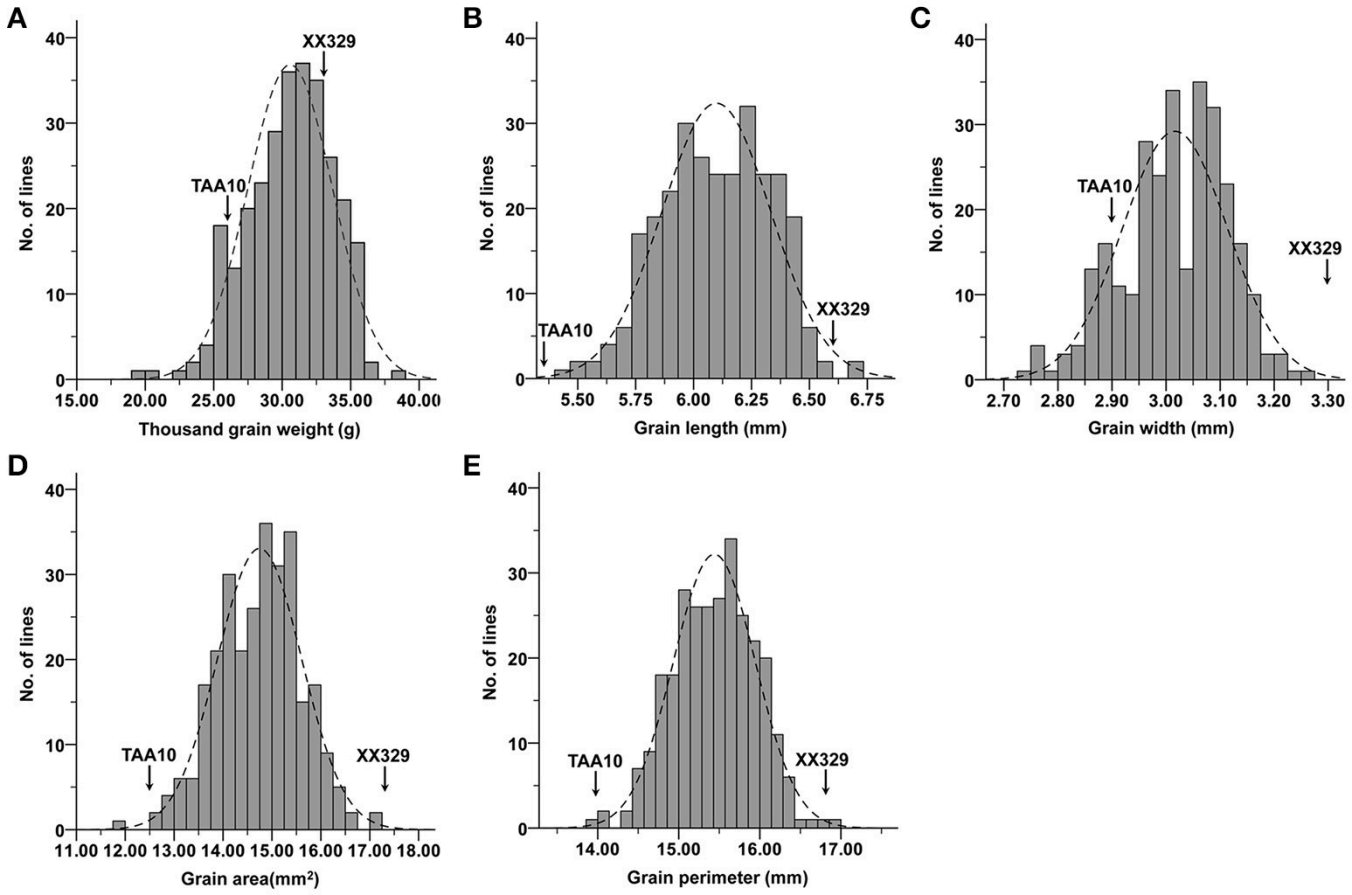
Frequency distributions for means of **(A)** thousand grain weight (TGW), **(B)** grain length (GL), **(C)** grain width (GW), **(D)** grain area (GA), and **(E)** grain perimeter (GP) in the F_2:3_ population.

The ETW had lower TGW, GL, GW, GA, and GP compared with TAA10, and these values of the resynthesized wheat XX329 were higher than those of TAA10 (Figure 1; Table 1). TGW, GL, GW, GA, and GP variations of the two parents (TAA10 and XX329) and the segregation populations were evaluated in six environments (Appendix B in Supplementary Material). XX329 consistently showed higher values by at least 6.65 g TGW, 1.17 mm GL, 0.13 mm GW, 2.58 mm^2^ GA, and 1.65 mm GP compared with TAA10 in all environments (Table 2). The frequency distributions of the investigated traits revealed continuous variations in the F_2:3_ population, suggesting that the phenotypic data of TGW, GL, GW, GA, and GP are normally distributed and these traits are controlled by multiple loci (Figure 2). Additionally, correlation coefficients among the TGW, GL, GW, GA, and GP traits in the F_2:3_ population were calculated. All five traits showed significant positive correlations with each other (Table 3). The strongest positive correlation was observed between GL and GP, followed by GA and GP. However, GL had a weak positive correlation with GW.

**Table 1:** Means and standard deviations of TAA10, ETW, TQ18 and XX329 for thousand grain weight (TGW), grain length (GL), grain width (GW), grain area (GA), and grain perimeter (GP).

	**TGW (g)**	**GL (mm)**	**GW (mm)**	**GA (mm^2^)**	**GP (mm)**
TAA10	26.22 ± 0.56^b^	5.17 ± 0.03^b^	2.81 ± 0.05^b^	11.57 ± 0.29^b^	13.30 ± 0.14^b^
ETW	13.90 ± 0.27^c^	4.59 ± 0.01^d^	2.25 ± 0.02^d^	8.32 ± 0.07^d^	11.66 ± 0.05^d^
TQ18	10.75 ± 0.94^d^	5.03 ± 0.14^c^	2.58 ± 0.03^c^	10.18 ± 0.38^c^	12.90 ± 0.31^c^
XX329	36.26 ± 1.22^a^	6.45 ± 0.10^a^	2.99 ± 0.04^a^	14.93 ± 0.39^a^	15.88 ± 0.22^a^

*Values are means ± standard deviations*.

*Different letters denote significant differences (P < 0.05) as determined by analysis of variance in each column*.

**Table 2:** Parental and population means, standard deviations, and ranges for thousand grain weight (TGW), grain length (GL), grain width (GW), grain area (GA), and grain perimeter (GP) in six environments.

**Environment**		**TGW (g)**	**GL (mm)**	**GW (mm)**	**GA (mm^2^)**	**GP (mm)**
Beijing2015	TAA10	26.15 ± 1.83	5.42 ± 0.22	2.86 ± 0.05	12.48 ± 0.83	13.98 ± 0.66
	XX329	33.11 ± 1.02^**^	6.59 ± 0.15^**^	3.29 ± 0.02^**^	17.3 ± 0.56^**^	16.8 ± 0.58^**^
	F_2:3_ population	30.58 ± 3.1	6.1 ± 0.23	3.02 ± 0.1	14.75 ± 0.86	15.44 ± 0.51
	Range in F_2:3_	19.75 – 38.24	5.44 – 6.72	2.73 – 3.27	11.83 – 17.20	13.87 – 16.90
	F_2_ population	27.17 ± 5.65	5.97 ± 0.28	2.88 ± 0.21	13.89 ± 1.46	15.06 ± 0.72
	Range in F_2_	11.44 – 42.30	4.67 – 6.71	2.28 – 3.34	9.71 – 18.12	12.17 – 17.15
Beijing2016	TAA10	28.64 ± 0.08	5.5 ± 0.07	3 ± 0.02	13.03 ± 0.14	14.26 ± 0.14
	XX329	39.31 ± 1.46^**^	6.74 ± 0.09^**^	3.21 ± 0.02^**^	16.91 ± 0.28^**^	16.92 ± 0.14^**^
	F_2_ population	29.07 ± 4.77	5.86 ± 0.34	2.88 ± 0.17	13.52 ± 1.38	14.91 ± 0.86
	Range in F_2_	12.00 – 45.00	4.64 – 7.06	2.38 – 3.27	8.84 – 17.63	11.85 – 19.22
Hebei2015	TAA10	25.56 ± 0.56	5.16 ± 0.16	2.69 ± 0.03	10.77 ± 0.31	12.13 ± 0.12
	XX329	32.21 ± 0.8^**^	6.35 ± 0.05^**^	3.22 ± 0.03^**^	17.08 ± 0.12^**^	16.2 ± 0.03^**^
	F_2_ population	23.77 ± 5.62	5.87 ± 0.28	2.97 ± 0.22	14.12 ± 1.45	15.14 ± 0.69
	Range in F_2_	8.39 – 35.63	4.80 – 6.57	2.30 – 3.40	9.40 – 17.78	12.13 – 16.87
Hebei2016	TAA10	29.56 ± 0.43	5.37 ± 0.01	3.07 ± 0.02	13.09 ± 0.14	14.1 ± 0.02
	XX329	41.48 ± 0.95^**^	6.74 ± 0.16^**^	3.25 ± 0.02^**^	16.92 ± 0.58^**^	16.94 ± 0.41^**^
	F_2_ population	31.11 ± 6.19	5.77 ± 0.25	2.92 ± 0.23	13.49 ± 1.38	14.78 ± 0.7
	Range in F_2_	10.00 – 40.91	4.47 – 6.32	2.04 – 3.30	7.64 – 16.06	11.31 – 16.03
Shanxi2015	TAA10	27.56 ± 0.55	5.3 ± 0.02	3.03 ± 0.01	12.08 ± 0.22	13.92 ± 0.1
	XX329	35.55 ± 0.82^**^	6.63 ± 0.1^**^	3.16 ± 0.05^*^	16.43 ± 0.18^**^	15.86 ± 0.1^**^
	F_2_ population	27.54 ± 5.13	5.79 ± 0.33	2.97 ± 0.19	13.96 ± 1.49	14.86 ± 0.8
	Range in F_2_	13.85 – 40.50	4.81 – 6.53	2.38 – 3.40	9.56 – 18.02	12.49 – 16.92
Shanxi2016	TAA10	26.89 ± 0.64	5.18 ± 0.25	2.97 ± 0.02	12.57 ± 0.16	13.74 ± 0.08
	XX329	34.96 ± 0.82^**^	6.52 ± 0.13^**^	3.06 ± 0.08	15.15 ± 0.14^**^	15.39 ± 0.18^**^
	F_2_ population	30.58 ± 4.66	5.85 ± 0.33	3.03 ± 0.17	14.16 ± 1.37	15.07 ± 0.78
	Range in F_2_	18.20 – 44.63	4.81 – 6.57	2.51 – 3.59	10.22 – 18.68	12.57 – 17.30

*Values are means ± standard deviations*.

*, ** *Indicate significant differences in phenotypic values between the parents by t-test at the 0.05 and 0.01 levels, respectively*.

**Table 3:** Correlation coefficients among thousand grain weight (TGW), grain length (GL), grain width (GW), grain area (GA), and grain perimeter (GP) in the F_2:3_ population.

**Trait**	**TGW**	**GL**	**GW**	**GA**
GL	0.70^**^			
GW	0.70^**^	0.37^**^		
GA	0.87^**^	0.85^**^	0.79^**^	
GP	0.76^**^	0.96^**^	0.59^**^	0.95^**^

** *Indicates significance at the 0.01 level*.

In the plot-yield trials, XX329 had higher TGW than TAA10 in the two environments. Conversely, grain number per spike of TAA10 was significantly higher than that of XX329. No significant differences were observed in spike length, spikelet number per spike, and spike number per plant. The grain yield per plot of XX329 increased by 17.43–30.36% compared with that of TAA10 in two environments (Table 4).

**Table 4:** Means and standard deviations for spike length (SL), spikelet number of per spike (SLN), spike number per plant (SN), grain number per spike (GN), thousand grain weight (TGW), and yield per plot (YPP) in plot-yield trials of TAA10 and XX329 under two environments.

**Environment**		**SL (cm)**	**SLN**	**SN**	**GN**	**TGW (g)**	**YPP (kg)**
Beijing2015	TAA10	13.90 ± 1.37	21.73 ± 1.31	32.23 ± 5.93	44.17 ± 0.86^**^	27.88 ± 2.04	16.57 ± 2.37
	XX329	13.25 ± 1.23	21.10 ± 1.42	31.60 ± 6.18	38.23 ± 0.66	44.00 ± 1.70^**^	21.60 ± 1.06^*^
Hebei2015	TAA10	11.27 ± 0.32	20.33 ± 0.39	30.17 ± 4.19	46.26 ± 0.87^**^	29.20 ± 0.56	16.35 ± 1.34
	XX329	10.91 ± 0.10	20.13 ± 0.49	28.67 ± 3.62	37.17 ± 1.13	43.74 ± 0.80^**^	19.20 ± 0.41^*^

*Values are means ± standard deviations*.

*, ** *Indicate significant differences in phenotypic values between the parents by t-test at the 0.05 and 0.01 levels, respectively*.

### SNP based genetic difference between TAA10 and XX329

To evaluate the genetic difference between TAA10 and XX329, wheat 660K SNP chip with 630,517 makers was employed for analysis (Appendix D in Supplementary Material). Of 594,299 SNP markers with genotype data, 48,753 (8.20%) markers exhibited polymorphism between TAA10 and XX329. Based on genetic position information provided by Compass Biotechnology Company, 392,088 of 594,299 SNP markers have been mapped to wheat genomes, and the numbers on A, B, and D genomes were 148,425, 179,667, and 63,996, respectively. Accordingly, 30,121 of 48,753 polymorphic SNP markers between TAA10 and XX329 could be mapped to wheat genomes, and the numbers on A, B, and D genomes were 5,114, 3,417, and 21,590 markers, respectively ((Table S1). Taken together, the genetic similarities of A, B, and D genomes between TAA10 and XX329 were 96.55, 98.10, and 66.26%, respectively. Notably, 2,617 of 5,114 polymorphic SNP markers on A genome were located on chromosome 1A, among which the majority (1988, 75.96%) was located in the telomere region on the long arm of chromosome 1A.

### Single marker analysis

A total of 436 D-genomic SSR markers were screened, and 81 polymorphic markers between TAA10 and XX329 were used for single marker analysis of TGW, GL, GW, GA, and GP by genotyping 328 individuals from the F_2_ population grown in 2014 and using the phenotypic data of the 328 F_2:3_ lines grown in 2015. The results showed that two markers *Xbarc11* and *Xcfd2* on chromosome 2D were significantly associated with all five traits, and four markers (*Xgwm261, Xcfd53* on chromosome 2D and *Xwmc702, Xbarc260* on chromosome 7D) were significantly linked with GL, GA, and GP (Table 5).

**Table 5:** Single marker analysis of markers on chromosomes 2D and 7D with thousand grain weight (TGW), grain length (GL), grain width (GW), grain area (GA). and grain perimeter (GP) in the F_2:3_ populations.

**Chromosome**	**Marker**	**TGW**			**GL**			**GW**			**GA**			**GP**		
		***F*_(1, n−2)_**	**Pr(F)**	***R*^2^**	***F*_(1, n−2)_**	**Pr(F)**	***R*^2^**	***F*_(1, n−2)_**	**Pr(F)**	***R*^2^**	***F*_(1, n−2)_**	**Pr(F)**	***R*^2^**	***F*_(1, n−2)_**	**Pr(F)**	***R*^2^**
2D	*Xbarc11*	32.03	0.0000^****^	0.10	39.85	0.0000^****^	0.14	44.96	0.0000^****^	0.13	81.14	0.0000^****^	0.22	67.30	0.0000^****^	0.19
	*Xcfd2*	44.09	0.0000^****^	0.14	45.23	0.0000^****^	0.15	64.76	0.0000^****^	0.19	92.34	0.0000^****^	0.25	75.04	0.0000^****^	0.22
	*Xgwm261*	2.12	0.1463	0.01	55.17	0.0000^****^	0.16	7.40	0.0069^**^	0.03	9.41	0.0023^**^	0.03	29.71	0.0000^****^	0.10
	*Xcfd53*	2.39	0.1231	0.01	65.01	0.0000^****^	0.19	7.51	0.0065^**^	0.03	11.93	0.0006^***^	0.04	36.05	0.0000^****^	0.11
7D	*Xwmc702*	3.35	0.6812	0.01	36.77	0.0000^****^	0.11	1.94	0.1645	0.01	17.64	0.0000^****^	0.06	32.11	0.0000^****^	0.10
	*Xbarc260*	5.19	0.0234^*^	0.02	28.05	0.0000^****^	0.09	0.01	0.9799	0.00	8.86	0.0031^**^	0.03	18.44	0.0000^****^	0.06

*, **, ***, and **** *Indicate significance at the 0.05, 0.01, 0.001, and 0.0001 levels, respectively*.

### Linkage map construction and QTL analysis

**Figure 3:**
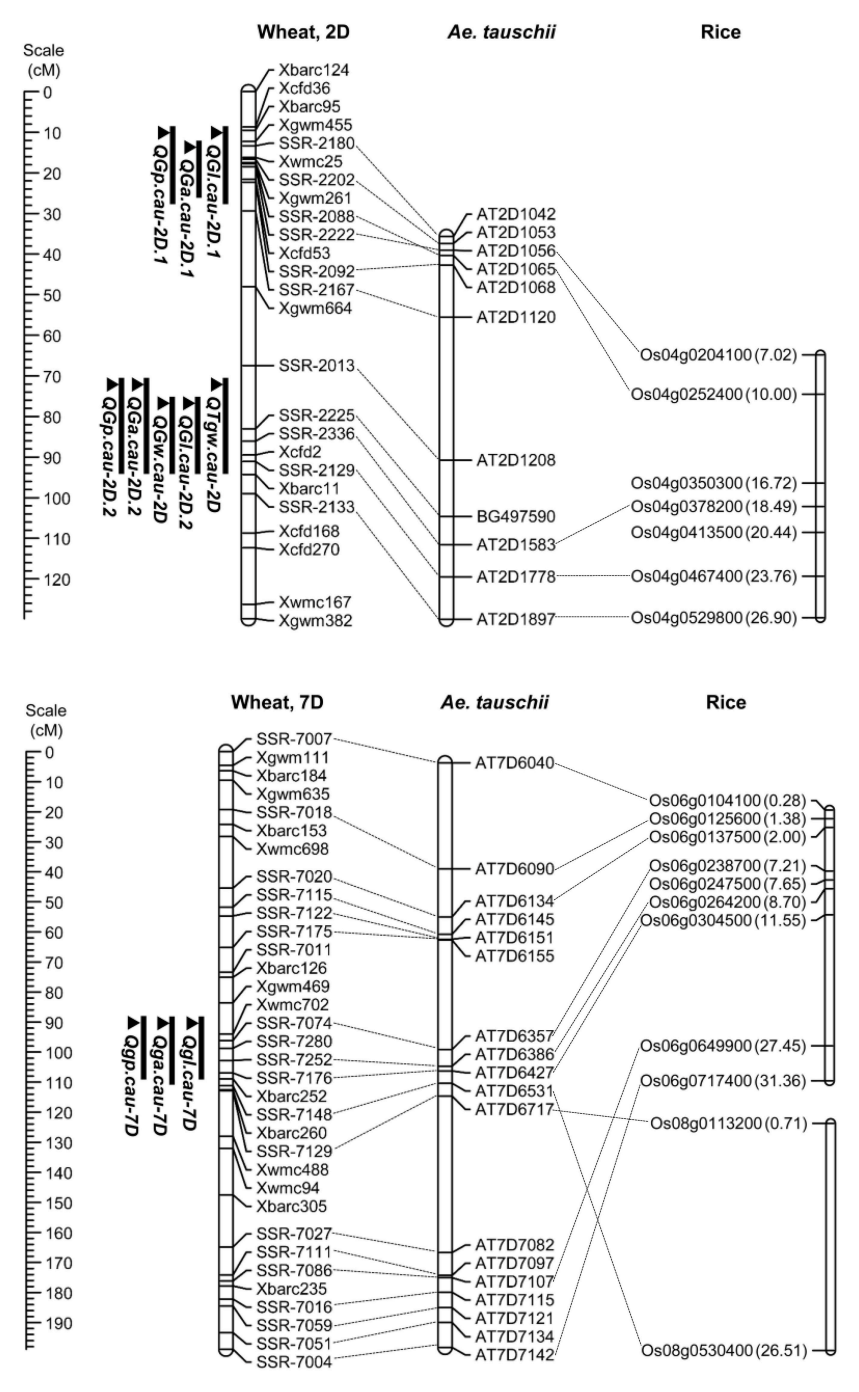
Locations of the detected QTL on the chromosome 2D and 7D in the F_2:3_ population and the collinearity of the developed markers, *Ae. Tauschii* markers and rice genes. A centiMorgan (cM) scale is shown on the left. The black ellipse represents the approximate location of the centromere. Vertical bars show the confidence intervals for the location of each QTL with LOD from the top to 3.0.

To further conduct QTL analysis for traits related to grain size and shape on chromosomes 2D and 7D, 31 SSR makers were developed using the referential sequence from *Ae. tauschii*, and the information of developed SSR markers for linkage map construction was shown in Appendix C in Supplementary Material. The collinearities of these markers with the *Ae. tauschii* markers and rice genes were shown in Figure 3. These polymorphic markers were used for the linkage analysis by genotyping 328 individuals from the F_2_ population grown in 2014 (Appendix A in Supplementary Material). The resulting linkage maps of chromosomes 2D and 7D consisted of 25 and 34 SSR markers, spanning 129.78 and 198.88 cM in length, respectively (Figure 3).

Using the phenotypic data from the 328 F_2:3_ lines grown in 2015, 11 QTL located on chromosomes 2D and 7D were identified for the five grain traits (Table 6). One QTL associated with TGW was located on the long arm of chromosome 2D, which explained 13.91% of the phenotypic variation, and it was designated *QTgw.cau-2D*. XX329 contributed effects for increased TGW at the QTL. Additionally, one QTL for GW (*QGw.cau-2D*) was detected in the same QTL interval on chromosome 2DL and explained 18.1% of the GW variation. The QTL associated with GL, GA and GP were all identified in each of the three genomic regions, chromosome 2DS (*QGl.cau-2D.1, QGa.cau-2D.1*, and *QGp.cau-2D.1*), chromosome 2DL (*QGl.cau-2D.2, QGa.cau-2D.2*, and *QGp.cau-2D.2*) and chromosome 7DS (*QGl.cau-7D, QGa.cau-7D*, and *QGp.cau-7D*). Three QTL for GL explained the proportions of phenotypic variation ranging from 10.50 to 20.13%, accordingly from 4.65 to 25.12% for GA and from 10.05 to 12.40% for GP. XX329 contributed favorable alleles at all of the identified QTL (Figure 3; Table 6).

**Table 6:** A summary of QTL for thousand grain weight (TGW), grain length (GL), grain width (GW), grain area (GA), and grain perimeter (GP) in the F_2:3_ population.

**Chromosome**	**Marker interval**	**Included QTL**	**Position (cM)^a^**	**LOD**	**Additive effect^b^**	**Contribution (%)**
2DS	*Xbarc95-Xcfd53*	*QGl.cau-2D.1*	9.5	10.9	−0.17	20.13
		*QGa.cau-2D.1*	7.8	3.8	−0.33	4.65
		*QGp.cau-2D.1*	9.5	13.3	−0.33	12.40
2DL	*SSR-2013-Xbarc11*	*QTgw.cau-2D*	17.5	11.0	−1.68	13.91
		*QGl.cau-2D.2*	15.4	3.7	−0.08	10.50
		*QGw.cau-2D*	20.8	4.8	−0.05	18.63
		*QGa.cau-2D.2*	17.5	9.1	−0.44	25.12
		*QGp.cau-2D.2*	17.5	7.7	−0.23	10.05
7DS	*Xwmc702-Xbarc252*	*QGl.cau-7D*	7.3	17.1	−0.15	13.13
		*QGa.cau-7D*	7.3	8.5	−0.43	5.87
		*QGp.cau-7D*	4.9	15.2	−0.32	10.18

a *Position for the QTL is the distance from the first marker in the interval to the peak value of the QTL*.

b *Positive and negative effects indicate that the TAA10 and XX329 alleles increased value for each trait, respectively*.

### Environmental stability assessment of the QTL

To further investigate the environmental stability of the 11 QTL, another six F_2_ populations derived from TAA10 and XX329 were grown at three locations (Beijing, Hebei and Shanxi) during 2 years (2014 and 2015). Three SSR markers (*Xgwm455, Xgwm261*, and *Xcfd53*) on chromosome 2DS, four markers (*SSR-2225, SSR-2336, SSR-2129*, and *Xbarc11*) on chromosome 2DL and six markers (*Xwmc702, SSR-7074, SSR-7252, SSR-7176, Xbarc260*, and *Xwmc488*) on chromosome 7DS in and nearby the QTL intervals were used for linkage map construction by genotyping individuals from each F_2_ population. The QTL that could be detected in five or more environments were regarded as “environmentally stable QTL.” According to this criterion, nine of them were environmentally stable QTL. In the QTL interval on chromosome 2DS, *QGl.cau-2D.1, QGa.cau-2D.1*, and *QGp.cau-2D.1* were detected in all six F_2_ populations, which explained from 14.84 to 32.78%, from 3.70 to 20.03%, and from 8.91 to 27.43% of phenotypic variation, respectively. Remarkably, *QTgw.cau-2D, QGl.cau-2D.2, QGw.cau-2D, QGa.cau-2D.2*, and *QGp.cau-2D.2* in the region on chromosome 2DL were all environmentally stable in the six F_2_ populations, and the QTL for TGW, GL, and GW explained the proportion of phenotypic variation ranging from 4.28 to 13.72%, from 5.93 to 21.92%, and from 3.59 to 10.48%, respectively. In addition, among the QTL on chromosome 7DS, *QGl.cau-7D* was the environmentally stable QTL detected in five environments and explained from 3.80 to 12.30% of the GL variation (Table 7).

**Table 7:** A summary of QTL for thousand grain weight (TGW), grain length (GL), grain width (GW), grain area (GA), and grain perimeter (GP) in the six F_2_ populations.

**Chromosome**	**Population**	**Marker interval**	**Included QTL**	**Position (cM)^a^**	**LOD**	**Additive effect^b^**	**Contribution (%)**
2DS	F_2_ (Beijing2015)	*Xgwm455-Xcfd53*	*QGl.cau-2D.1*	9.6	19.6	−0.27	15.65
			*QGa.cau-2D.1*	9.6	4.6	−0.63	3.70
			*QGp.cau-2D.1*	9.6	11.2	−0.44	8.91
	F_2_ (Beijing2016)	*Xgwm455-Xcfd53*	*QGl.cau-2D.1*	5.5	10.5	−0.26	20.15
			*QGa.cau-2D.1*	5.5	3.4	−0.64	6.67
			*QGp.cau-2D.1*	5.5	6.5	−0.54	13.35
	F_2_ (Hebei2015)	*Xgwm455-Xcfd53*	*QGl.cau-2D.1*	8.3	9.8	−0.23	14.84
			*QGa.cau-2D.1*	8.3	2.7	−0.59	5.50
			*QGp.cau-2D.1*	8.3	6.1	−0.39	10.08
	F_2_ (Hebei2016)	*Xgwm455-Xcfd53*	*QGl.cau-2D.1*	7.2	9.8	−0.22	19.25
			*QGa.cau-2D.1*	7.2	2.2	−0.56	4.36
			*QGp.cau-2D.1*	7.2	3.9	−0.41	9.61
	F_2_ (Shanxi2015)	*Xgwm455-Xcfd53*	*QGl.cau-2D.1*	18.1	10.8	−0.26	24.36
			*QGa.cau-2D.1*	18.1	4.1	−0.74	10.10
			*QGp.cau-2D.1*	18.1	6.7	0.64	16.90
	F_2_ (Shanxi2016)	*Xgwm455-Xcfd53*	*QGl.cau-2D.1*	12.9	24.7	−0.30	32.78
			*QGa.cau-2D.1*	12.9	10.7	−1.03	20.03
			*QGp.cau-2D.1*	12.9	17.0	−0.76	27.43
2DL	F_2_ (Beijing2015)	*SSR-2336-Xbarc11*	*QTgw.cau-2D*	3.6	4.3	−2.31	4.41
			*QGl.cau-2D.2*	3.6	13.3	−0.18	11.38
			*QGw.cau-2D*	3.6	3.5	−0.08	3.73
			*QGa.cau-2D.2*	3.6	8.1	−0.76	8.37
			*QGp.cau-2D.2*	4.2	11.2	−0.43	11.11
	F_2_ (Beijing2016)	*SSR-2225-Xbarc11*	*QTgw.cau-2D*	5.0	3.2	−2.14	6.79
			*QGl.cau-2D.2*	6.5	7.8	−0.24	16.93
			*QGw.cau-2D*	5.0	3.0	−0.08	5.68
			*QGa.cau-2D.2*	5.0	6.6	−0.89	12.98
			*QGp.cau-2D.2*	5.0	6.2	−0.51	12.36
	F_2_ (Hebei2015)	*SSR-2225-Xbarc11*	*QTgw.cau-2D*	9.8	2.2	−1.75	4.28
			*QGl.cau-2D.2*	7.8	2.6	−0.11	5.93
			*QGw.cau-2D*	9.8	2.3	−0.08	4.11
			*QGa.cau-2D.2*	7.8	4.1	−0.51	6.61
			*QGp.cau-2D.2*	7.8	3.6	−0.35	7.65
	F_2_ (Hebei2016)	*SSR-2225-Xbarc11*	*QTgw.cau-2D*	6.7	2.5	−2.19	5.23
			*QGl.cau-2D.2*	7.9	3.1	−0.10	8.60
			*QGw.cau-2D*	6.7	2.1	−0.07	3.59
			*QGa.cau-2D.2*	6.7	3.1	−0.54	7.72
			*QGp.cau-2D.2*	7.9	3.4	−0.29	8.33
	F_2_ (Shanxi2015)	*SSR-2225-Xbarc11*	*QTgw.cau-2D*	8.4	6.0	−3.00	13.72
			*QGl.cau-2D.2*	5.3	7.3	−0.20	21.92
			*QGw.cau-2D*	8.4	6.0	−0.12	10.48
			*QGa.cau-2D.2*	8.4	7.5	−1.03	17.72
			*QGp.cau-2D.2*	8.4	7.6	−0.56	15.72
	F_2_ (Shanxi2016)	*SSR-2225-SSR-2129*	*QTgw.cau-2D*	2.9	2.4	−1.72	6.95
			*QGl.cau-2D.2*	2.9	3.1	−0.11	10.72
			*QGw.cau-2D*	2.9	2.6	−0.06	6.68
			*QGa.cau-2D.2*	2.9	3.7	−0.57	10.44
			*QGp.cau-2D.2*	2.9	3.3	−0.29	10.85
7DS	F_2_ (Beijing2015)	*Xwmc702-SSR-7176*	*QGl.cau-7D*	4.3	5.6	−0.15	5.03
			*QGa.cau-7D*	4.3	2.1	−0.46	3.25
			*QGp.cau-7D*	4.3	4.0	−0.31	4.76
	F_2_ (Hebei2015)	*Xwmc702-SSR-7252*	*QGl.cau-7D*	4.0	2.6	−0.10	3.80
	F_2_ (Hebei2016)	*Xwmc702-SSR-7176*	*QGl.cau-7D*	0.1	4.9	−0.15	8.67
			*QGp.cau-7D*	0.1	2.2	−0.23	4.60
	F_2_ (Shanxi2015)	*Xwmc702-SSR-7176*	*QGl.cau-7D*	7.4	7.0	−0.19	12.30
			*QGa.cau-7D*	4.5	3.3	−0.67	6.21
			*QGp.cau-7D*	7.4	5.0	−0.40	9.33
	F_2_ (Shanxi2016)	*Xwmc702-SSR-7176*	*QGl.cau-7D*	14.0	4.4	−0.23	12.30
			*QGa.cau-7D*	14.0	3.6	−0.48	5.48
			*QGp.cau-7D*	14.0	3.8	−0.29	9.05

a *Position for the QTL is the distance from the first marker in the interval to the peak value of the QTL*.

b *Positive and negative effects indicate that the TAA10 and XX329 alleles increased value for each trait, respectively*.

### QTL verification using BC_4_F_2_ populations

To further verify the QTL on chromosomes 2DS, 2DL, and 7DS, five NIL populations were developed. In the process of backcross, the SSR markers in the three QTL regions were used for foreground selection: five markers (X*cfd36, Xgwm455, Xgwm261, Xcfd53*, and *SSR-2092*) for the 2DS region, six markers (*SSR-2225, SSR-2336, Xcfd2, SSR-2129, Xbarc11*, and *SSR-2133*) for the 2DL region and six markers (*Xwmc702, SSR-7074, SSR-7252, SSR-7176, Xbarc252*, and *SSR-7148*) for the 7DS region. Finally, five BC_4_F_1_ individuals (BC_4_F_2_-2DS, BC_4_F_2_-2DL-1, BC_4_F_2_-2DL-2, BC_4_F_2_-7DS-1, and BC_4_F_2_-7DS-2) were self-pollinated to produce their corresponding BC_4_F_2_ populations, which exhibited heterozygosity in corresponding QTL region and 93.97 to 98.31% similarities in genetic background with the recurrent parent (Appendix E in Supplementary Material).

To determine whether the identified QTL affect the traits of grain size and shape in each BC_4_F_2_ population, we compared TGW, GL, GW, GA, and GP traits between two homozygous groups, that is, TAA10 homozygotes and XX329 homozygotes (Table 8; Appendix F in Supplementary Material). In the BC_4_F_2_-2DS population, the mean values of GL, GA and GP of the XX329 homozygotes showed significantly higher than those of TAA10 homozygotes, whereas TGW and GW had no significant differences between the two homozygous groups. In the two BC_4_F_2_-2DL-1 and BC_4_F_2_-2DL-2 populations, the XX329 homozygous groups exhibited significantly higher TGW, GL, GW, GA, and GP than TAA10. In addition, the significant differences of GL, GA, and GP were identified between TAA10 and XX329 homozygous groups in the BC_4_F_2_-7DS-1 population, and the significant differences of GL and GP were found between two homozygous groups in the BC_4_F_2_-7DS-2 population (Table 8). Collectively, these results provided further evidence that these QTL on chromosomes 2D and 7D significantly affected on grain size and shape, which were in agreement with the results by the F_2_ and F_2:3_ population.

**Table 8:** Variation between two homozygous groups of five NIL populations for thousand grain weight (TGW), grain length (GL), grain width (GW), grain area (GA), and grain perimeter (GP).

**Population**	**TGW (g)**		**GL (mm)**		**GW (mm)**		**GA (mm**^**2**^**)**		**GP (mm)**	
	**TT^a^**	**XX^b^**	**TT**	**XX**	**TT**	**XX**	**TT**	**XX**	**TT**	**XX**
BC_4_F_2_-2DS	29.94 ± 2.82	30.87 ± 4.21	5.40 ± 0.18	5.72 ± 0.20^****^	2.87 ± 0.16	2.88 ± 0.15	12.42 ± 0.87	13.16 ± 0.96^***^	14.38 ± 0.65	15.06 ± 0.67^****^
BC_4_F_2_-2DL-1	28.86 ± 3.73	32.72 ± 3.11^***^	5.49 ± 0.25	5.75 ± 0.46^*^	2.88 ± 0.20	2.99 ± 0.17^*^	12.65 ± 1.23	13.73 ± 1.77^*^	14.58 ± 0.80	15.21 ± 0.83^**^
BC_4_F_2_-2DL-2	28.50 ± 4.61	33.26 ± 2.90^***^	5.34 ± 0.20	5.75 ± 0.27^****^	2.75 ± 0.14	2.96 ± 0.17^****^	11.70 ± 0.86	13.58 ± 1.15^****^	14.36 ± 0.73	15.25 ± 0.55^****^
BC_4_F_2_-7DS-1	30.30 ± 3.76	31.93 ± 3.86	5.38 ± 0.22	5.69 ± 0.22^****^	2.93 ± 0.14	2.94 ± 0.15	12.63 ± 0.97	13.34 ± 1.03^*^	14.52 ± 0.85	15.11 ± 0.87^*^
BC_4_F_2_-7DS-2	29.63 ± 3.04	30.98 ± 3.15	5.45 ± 0.18	5.72 ± 0.23^***^	2.91 ± 0.10	2.88 ± 0.14	12.68 ± 0.73	13.14 ± 1.01	14.55 ± 0.63	15.02 ± 0.63^*^

a *TT represents TAA10 homozygote*.

b *XX represents XX329 homozygote*.

*Values are means ± standard deviations*.

*, **, ***, and **** *Indicate significant differences in values between the two homozygous groups by t-test at the 0.05, 0.01, 0.001, and 0.0001 levels, respectively*.

## Discussion

### SNP marker-based genetic similarity between TAA10 and XX329

Resynthesized allohexaploid wheat XX329 (AABBDD) was produced by crossing ETW (AABB) and the *Ae. tauschii* subsp *strangulate* (TQ18, DD). ETW contains an AABB genome from natural allohexaploid bread wheat donor (TAA10) by nine cycles of backcrossing (Kerber, 1964). Theoretically, the genome (AABB) of XX329 should be >99.8% identical to the AABB subgenomes of its bread wheat donor (TAA10) after the ninth backcross (Zhang et al., 2014). To verify this hypothesis, wheat 660K SNP chip was firstly employed for analysis in this study. Based on the information of SNP markers with genetic position, we found that the A and B genomes of TAA10 and XX329 showed 96.55 and 98.10% genetic similarities, respectively, which provided molecular evidence that the genome (AABB) of XX329 was near-identical to that of TAA10. Interestingly, we observed that the numbers of polymorphic SNP markers are not uniform across the A genome. Of 2617 polymorphic SNP markers on chromosome 1A, 1988 (75.96%) were located in the telomere region of the long arm. This phenomenon may be attributed to genetic recombination or genomic variation during the process of extracting the AABB genomes, which is needed for further investigation.

Allohexaploid common wheat (AABBDD) evolved by natural hybridization of emmer wheat (AABB) and *Aegilops tauschii Coss*. (DD; Nesbitt and Samuel, 1995; Petersen et al., 2006). Growing evidence revealed that a few *Ae. tauschii*'s intraspecific lineages contributed to the evolution of common wheat, which resulted in relatively narrow genetic variation on the D-genome in wheat (Dvorak et al., 1998; Dubcovsky and Dvorak, 2007). Consistently, Jin et al. (2016) and Cui et al. (2017) reported the construction of high-density genetic maps using the wheat 660K SNP chip based on recombinant inbred line populations derived from common allohexaploid wheats, and the number of polymorphic SNP markers on the D genome was 3,905 and 13,820, respectively, which were much lower than that of TAA10 and XX329. Remarkably, the resynthesized allohexaploid wheat XX329 manifested obvious different phenotypes at multiple growth/developmental stages relative to natural allohexaploid wheat TAA10 (Zhang et al., 2014). Considering the higher genetic similarity of A and B genomes between TAA10 and XX329, we proposed that the observed phenotypic variation was mainly induced by differences on the D genome between TAA10 and XX329.

### Contribution of the D genome to grain size and shape in allohexaploid wheat

Grain size and shape are important traits in wheat and represent a classical example of a trait with variations that arose after polyploidization and domestication. A long, thin primitive grain was transformed into a wider, shorter modern grain during wheat domestication, indicating that grain shape became rounder during wheat domestication (Gegas et al., 2010). A notable aspect of polyploid wheat evolution is genomic asymmetry in the control of grain shape, and that is the predominant control of grain shape by the A genome (Feldman et al., 2012). However, the wide variation in grain size and shape observed among *Ae. tauschii* genotypes is retained in the synthetic allohexaploid wheat using natural tetraploid species as the AB genome donor, suggesting that the D genome partially affects grain size and shape of allohexaploid wheat (Röder et al., 2008; Okamoto et al., 2013; Rasheed et al., 2014). Here, we found that the D genome could lead to drastic change in grain size and shape of allohexaploid wheat. First, the grain length, width, and size of the extracted allotetraploid wheat (ETW; AABB) are significantly reduced compared with the donor allohexaploid bread wheat (TAA10). Second, the grain length, width and size of the resynthesized allohexaploid wheat (XX329) obtained by crossing ETW and *Ae. tauschii* subsp *strangulate* (TQ18) are much higher than that of TAA10. Remarkably, although the increased kernel weight of XX329 is at the expense of reducing grain number per spike, the grain yield per plant of XX329 is significantly enhanced compared with TAA10. Collectively, these data indicated that synthetic allohexaploid wheats with the D genome from *Ae. tauschii* is a potentially useful resource for genetic improvement of yield in wheat breeding.

### A novel QTL controlling grain weight on chromosome 2DL from the synthetic allohexaploid wheat

Allohexaploid common wheat was produced by natural hybridization of emmer wheat and *Ae. tauschii Coss*. When compared with the A and B genome, relatively narrow genetic variation was detected on the D genome, which was partially attributed to a limited number of *Ae. tauschii* involved in the evolution of common wheat (Dubcovsky and Dvorak, 2007). Synthetic allohexaploid wheats (AABBDD) provide potentially novel genetic variations associated with the D genome of *Ae. tauschii*. To date, some useful genes/QTL controlling desirable traits have been identified on the D genome of synthetic allohexaploid wheat, including disease resistance, abiotic stress tolerance, suitable quality and anti-sprouting (Tadesse et al., 2007; Imtiaz et al., 2008; Li et al., 2012; Ilyas et al., 2015).

Grain weight is an important component of grain yield. Recently, Simmonds et al. (2014) reported that the effect of yield QTL, located on chromosome 6A, was driven primarily by increased grain weight, suggesting that the enhancement of grain weight could contribute to the genetic improvement of wheat yield. Several studies were conducted to identify beneficial QTL for grain weight from the diploid D donor of common wheat (Liao et al., 2008; Röder et al., 2008). For example, an environmentally stable QTL *QGw.caas-3D* was identified on chromosome 3D and the synthetic allohexaploid wheat Am3 contributed effect for increased grain weight (Liao et al., 2008). In this study, QTL mapping for grain weight was conducted using the F_2_ and F_2:3_ populations derived from TAA10 and XX329. One major QTL (*QTgw.cau-2D*), with the synthetic allohexaploid wheat XX329 contributing favorable alleles, was consistently detected on chromosome 2DL under different environments, which was linked to the marker *SSR-2336*. In the QTL region of *QTgw.cau-2D*, QTL for GL, GW, GA, and GP were also identified under different environments, and these co-localized QTL shared similar confidence intervals and had tightly linked QTL peak positions, which are indicative of potential pleiotropy among the traits. To the best of our knowledge, no QTL for grain weight has been detected on the long arm of chromosome 2D from synthetic allohexaploid wheats. Therefore, this QTL detected in our populations represent a novel loci controlling grain weight from the diploid D donor, which may be a more desirable target for genetic improvement in wheat breeding. However, grain weight is generally negatively correlated with grain number per spike and spike number per plant. Thus, it is necessary to develop lines carrying introgression segments with this region and to further clarify the function of *QTgw.cau-2D* to yield and its components in different genetic backgrounds.

### The effect of consensus QTL on chromosomes 2DS and 7DS to grain shape

**Figure 4:**
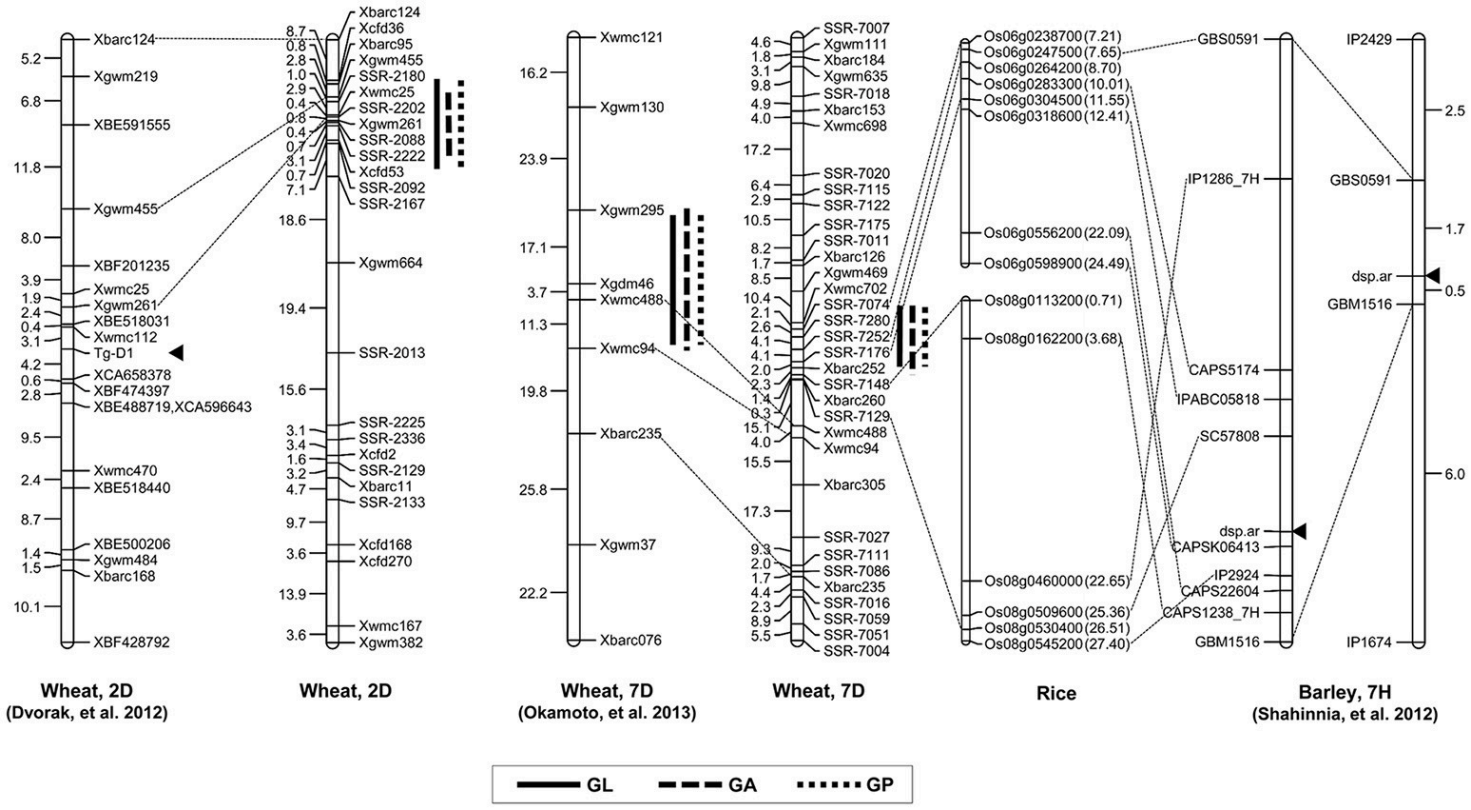
Comparative maps of QTL on chromosomes 2D and 7D. Vertical bars show the confidence intervals for the location of each QTL with LOD from the top to 3.0. The number in brackets indicates the physical position (Mb) of the rice gene.

Grain shape is a complex quantitative and important agronomic trait. To date, many studies have identified QTL controlling grain shape in common wheat cultivars, and these QTL were assigned to various chromosomes (Dholakia et al., 2003; Breseghello and Sorrells, 2006; Sun et al., 2009; Williams and Sorrells, 2014). Recently, genetic loci controlling the differences in grain shape between common wheat and synthetic hexaploids have been investigated (Okamoto et al., 2012; Yu et al., 2014). Glumes tenaciously enclose grains in the synthetic allohexaploid wheat lines, whereas modern cultivars are free-threshing wheat. Interestingly, one pleiotropic locus on chromosome 2DS significantly contributed to the determination of wheat grain shape, which corresponded to that of *Tg-D1* (Dvorak et al., 2012). Consistent with the result, we found that a major QTL with an LOD score of higher than 9.8 was located on the short arm of chromosome 2D and the allele from synthetic wheat at the QTL produced longer grains. The molecular markers *Xbarc95* and *Xcfd53* flanked this QTL at an interval of 12.02 cM, which was located in the same position as *Tg-D1* (Figure 4). Notably, no QTL was detected for grain width and weight on chromosome 2DS. Collectively, our data supported the notion that wheat grains were rapidly improved to the smaller, rounder phenotype that accompanied the formation of free-threshing wheat, because the domestication from *Tg1Tg1* to *tg1tg1* occurred at an early phase after allohexaploid wheat speciation (Kerber and Rowland, 1974).

In the pleiotropic QTL region associated to GL, GA and GP on chromosome 7DS, *QGl.cau-7D* was the environmentally stable locus with the highest LOD value under different environments. When comparing previous results, we found that the region on chromosome 7DS was similar to the QTL interval reported by Okamoto et al. (2013) (Figure 4). Analogously, the QTL on chromosome 7D identified in that study significantly contributed to the variation of grain size and shape in the F_2_ population derived from two synthetic allohexaploid wheat lines, and further analysis was needed to clarify the relationship of these QTL. Several QTL contributing to important agronomic traits were fine mapped based on orthologous regions across several grass species (Chen et al., 2007; Handa et al., 2008; Somyong et al., 2011). In the present study, we increased markers' saturation in the QTL interval on 7DS using the referential sequence from *Ae. tauschii*, and the comparative analysis demonstrated that the peak region of *QGl.cau-7D* was syntenic to rice chromosome 6 at 7.21–8.70 Mb. *Dense spike-ar (dsp.ar)* in barley controlling spike density and morphology was identified on chromosome 7H, and the genomic region exhibited highly conserved synteny with part of rice chromosomes 6 and 8 (Shahinnia et al., 2012), which is similar to our QTL interval on chromosome 7DS (Figure 4). However, the QTL in the present study may not correspond to the same gene governing the spike and grain development because there was no significant difference in spike density decided by spike length and spikelet number per spike between TAA10 and XX329.

### Candidate genes controlling wheat grain size and shape

With orthologous genes controlling similar phenotypes across many grass species, including wheat, rice, barley, and sorghum, comparative genomics has shown collinearities and provided a powerful tool for gene discovery in wheat (Valluru et al., 2014). In the present study, the genomic region of the QTL on chromosome 2DL harboring stable QTL for TGW, GL, GW, GA, and GP exhibited good collinearity with the genomic region of rice chromosome 4 (Figure 3). Furthermore, the peak region of *QTgw.cau-2D* was syntenic to rice chromosome 4 at 18.49–23.76 Mb, and one cloned gene *GIF1* (*Os04g0413500*) encoding a cell-wall invertase required for carbon partitioning during early grain filling, which was located at 20.44 Mb on rice chromosome 4 (Wang et al., 2008). In addition, the peak region of QTL for GL, GA and GP on chromosome 7DS was syntenic to rice chromosome 6 at 7.21–8.70 Mb in which one cloned gene *PFP*_β_(*Os06g0247500*) regulating carbon metabolism during grain filling was located at 7.65 Mb on rice chromosome 6 (Figure 3; Duan et al., 2016). These results suggest that the two QTL for grain size and shape identified could be orthologous genes to *GIF1* and *PFP*_β_, which deserve further research.

### The advantages and limitations of F_2_ and F_2:3_ populations for QTL mapping

The F_2_ and F_2:3_ populations were extensively utilized for identification of QTL for a number of agronomic traits such as yield-related traits (Lu et al., 2011; Zhang et al., 2012; Wang et al., 2015). The advantage of the F_2_ and F_2:3_ mapping populations was that the process of construction was convenient and fast. However, the F_2_ and F_2:3_ populations were temporary separation populations and required large population size to ensure the precision of mapping (Zhang et al., 2010). A trade-off would be to further validate these QTL by the secondary separation populations such as NIL and residual heterozygous line (RHL) populations. As expected, in the present study, the identified QTL associated with grain size and shape based on the F_2_ and F_2:3_ populations could be verified using the NIL populations. Notably, the linkage maps based on six different F_2_ populations derived from the same cross using the SSR markers exhibited different genetic distance, which is normal in consideration of different population sizes and limited numbers of markers. However, the identified QTL from different F_2_ populations for the same QTL shared similar QTL intervals and peak positions. Thus, the identified QTL based on the F_2_ and F_2:3_ populations could be regarded as the same QTL. Additionally, the same QTL detected in different F_2_ and F_2:3_ populations exhibited different values of additive effect and contribution, which could be partially caused by environmental factors such as the climate and field conditions under different environments.

## Author contributions

ZN and QS conceived the project; LY, FL, HX, and XZ carried out experiments; LY analyzed experimental results; LY, QS, and ZN wrote the manuscript; HZ helped to revise the manuscript. All authors have read and approved the final manuscript.

### Conflict of interest statement

The authors declare that the research was conducted in the absence of any commercial or financial relationships that could be construed as a potential conflict of interest.

## Supplementary material

Table S1 The genetic difference on 21 wheat chromosomes based on SNP genotyping between TAA10 and XX329.

Appendix A Genotypic data of the F_2_ populations.

Appendix B Phenotypic data of the F_2_ and F_2:3_ populations.

Appendix C Information of developed SSR markers on chromosomes 2D and 7D for genetic map construction.

Appendix D Genotypic data of TAA10 and XX329 based on wheat 660K SNP chip.

Appendix E Genotypic data of five BC_4_F_1_ individuals for background selection.

Appendix F Genotypic and phenotypic data of two homozygous groups in each NIL population.

## Abbreviations

QTL: Quantitative trait locus/loci
SSR: Simple sequence repeat
SNP: Single nucleotide polymorphism
NIL: Near isogenic line
PAGE: Polyacrylamide gel electrophoresis
PCR: Polymerase chain reaction
CIM: Composite interval mapping
LOD: Logarithm of the odds
TGW: Thousand grain weight
GL: Grain length
GW: Grain width
GA: Grain area
GP: Grain perimeter
SL: Spike length
SLN: Spikelet number per spike
SN: Spike number per plant
GN: Grain number per spike
YPP: Yield per plot.
