# Supplementary material for: Identification of QTL for Grain Size and Shape on the D Genome of Natural and Synthetic Allohexaploid Wheats with Near-Identical AABB Genomes
Source: Front Plant Sci. 2017 Oct 12;8:1705. doi: 10.3389/fpls.2017.01705 (PMC5643848; doi:10.3389/fpls.2017.01705)
Supplement: Table S1 — The genetic difference on 21 wheat chromosomes based on SNP genotyping between TAA10 and XX329. [file Table1.docx]

| **Table S1** The genetic difference on 21 wheat chromosomes based on SNP genotyping between TAA10 and XX329. | | | | | | | | | | | | | | | | | | | | | | | |  |
| --- | --- | --- | --- | --- | --- | --- | --- | --- | --- | --- | --- | --- | --- | --- | --- | --- | --- | --- | --- | --- | --- | --- | --- | --- |
| Chromosome | A genome | | | | | | |  | B genome | | | | | | |  | D genome | | | | | | | |
|  | 1A | 2A | 3A | 4A | 5A | 6A | 7A |  | 1B | 2B | 3B | 4B | 5B | 6B | 7B |  | 1D | 2D | 3D | 4D | 5D | 6D | 7D | |
| No. of total markers | 26194 | 25752 | 16883 | 24350 | 14061 | 17099 | 24086 |  | 19982 | 33786 | 43127 | 12766 | 32199 | 22303 | 15584 |  | 8518 | 9020 | 6508 | 6746 | 10956 | 8942 | 13306 | |
| No. of polymorphic markers | 2617 | 488 | 381 | 512 | 279 | 342 | 495 |  | 352 | 593 | 907 | 200 | 534 | 346 | 485 |  | 3365 | 2546 | 2619 | 2876 | 3262 | 2796 | 4126 | |
| Ratio of polymorphic markers (%) | 9.99 | 1.90 | 2.26 | 2.10 | 1.98 | 2.00 | 2.06 |  | 1.76 | 1.76 | 2.10 | 1.57 | 1.66 | 1.55 | 3.11 |  | 39.50 | 28.23 | 40.24 | 42.63 | 29.77 | 31.27 | 31.01 | |
|  | | | | | | | | | | | | | | | | | | | | | | | |  |
